# Knowledge and perception about climate change and human health: findings from a baseline survey among vulnerable communities in Bangladesh

**DOI:** 10.1186/s12889-016-2930-3

**Published:** 2016-03-15

**Authors:** Md Iqbal Kabir, Md Bayzidur Rahman, Wayne Smith, Mirza Afreen Fatima Lusha, Syed Azim, Abul Hasnat Milton

**Affiliations:** Centre for Clinical Epidemiology and Biostatistics, School of Medicine and Public Health, Faculty of Health and Medicine, The University of Newcastle, Newcastle, NSW 2308 Australia; National Institute of Preventive and Social Medicine, NIPSOM, Mohakhali, Dhaka Bangladesh; School of Public Health and Community Medicine, Faculty of Medicine, University of New South Wales, Sydney, Australia; Climate Change and Health Promotion Unit, Ministry of Health and Family Welfare, Dhaka, Bangladesh

**Keywords:** Climate change, Health, Knowledge, Perception, Adaptation, Bangladesh

## Abstract

**Background:**

Bangladesh is one of the countries most vulnerable to climate change (CC). A basic understanding of public perception on vulnerability, attitude and the risk in relation to CC and health will provide strategic directions for government policy, adaptation strategies and development of community-based guidelines. The objective of this study was to collect community-based data on peoples’ knowledge and perception about CC and its impact on health.

**Methods:**

In 2012, a cross-sectional survey was undertaken among 6720 households of 224 enumeration areas of rural villages geographically distributed in seven vulnerable districts of Bangladesh, with total population of 19,228,598. Thirty households were selected randomly from each enumeration area using the household listing provided by the Bangladesh Bureau of Statistics (BBS). Information was collected from all the 6720 research participants using a structured questionnaire. An observation checklist was used by the interviewers to collect household- and community-related information. In addition, we selected the head of each household as the eligible participant for an interview. Evidence of association between sociodemographic variables and knowledge of CC was explored by cross-tabulation and measured using chi-square tests. Logistic regression models were used to further explore the predictors of knowledge.

**Results:**

The study revealed that the residents of the rural communities selected for this study largely come from a low socioeconomic background: only 9.6 % had postsecondary education or higher, the majority worked as day labourer or farmer (60 %), and only 10 % earned a monthly income above BDT 12000 (equivalent to US $150 approx.). The majority of the participants (54.2 %) had some knowledge about CC but 45.8 % did not (*p* < 0.001). The majority of knowledgeable participants (*n* = 3645) felt excessive temperature as the change of climate (83.2 %). Among all the respondents (*n* = 6720), 94.5 % perceived change in climate and extreme weather events. Most of them (91.9 %) observed change in rainfall patterns in the last 10 years, and 97.8 % people think their health care expenditure increased after the extreme weather events. Age, educational qualification, monthly income, and occupation were significantly associated with the knowledge about climate change (*p* < 0.001). People with higher educational level or who live near a school were more knowledgeable about CC and its impact on health.

**Conclusions:**

The knowledge level about CC in our study group was average but the perception and awareness of CC related events and its impact on health was high. The most influential factor leading to understanding of CC and its impact on health was education. School-based intervention could be explored to increase peoples’ knowledge about CC and necessary health adaptation at community level.

**Electronic supplementary material:**

The online version of this article (doi:10.1186/s12889-016-2930-3) contains supplementary material, which is available to authorized users.

## Background

The adverse effect of climate change on human health has been recognized relatively late in the development of climate science and policy making [1, 2]. The recent Fifth Assessment Report (AR5) of the Intergovernmental Panel on Climate Change (IPCC) reinforces the need for societies to take adaptive actions to protect human health from the adverse consequences of climate change [3]. Climate sensitive health determinants and outcomes pose a threat to public health in Least Developed Countries (LDC) [4–6]. To protect health, highly strategic interventions for adaptation will be needed over the next 20–30 years in LDCs such as Bangladesh [7–9].

Climate change (CC) related health risk is a multidimensional and cross cutting issue. To date, a number of studies have suggested that peoples’ perception of and attitude towards CC risk is closely related to adaptative behaviour and mitigation action [10–12]. However, few such studies have been carried out in developing countries [13–16]. Bangladesh is on top of the IPCC risk index of climate victims since 2007 [17].

In this context, epidemiological research has a major role to play in combating CC-related adverse health effects. In a recent cross-sectional study from two villages, one from the northern and another from the southern part of Bangladesh, households’ (*n* = 450) perception of human health risks were explored, which suggested the need for further research with a larger sample size to allow generalization [18]. Another study revealed that the public perceptions towards CC, and its impact on health, may inform policies to cope with associated health challenges of CC [19]. In some studies, lay people perceived CC with misconceptions and misunderstandings about its cause and effects [20–22]. These misunderstandings have the propensity to cause fear about the consequences of CC [23]. However, environmental change management and associated education from the government are important to build resilience among the vulnerable communities [24].

Knowledge of CC is a necessary precursor for people to adapt appropriately. The health sector of Bangladesh does not have evidence-based strategies for health adaptation to CC. A survey undertaken by the Ministry of Health and Family Welfare of Bangladesh forms the basis of a future cohort study. Such a study is needed to expand our knowledge of evidence-based translational research, particularly in the context of being one of the most vulnerable LDCs. The objective of this study was to collect baseline data on knowledge and perception of CC, and the awareness of prevalence of climate sensitive diseases, in selected vulnerable areas of Bangladesh. We also collected baseline data on CC-related diseases such as malaria, Dengue fever, pneumonia & diarrhoeal diseases. This paper focuses on knowledge and perception.

The results of this study provide useful information for policy makers and other stakeholders in the health sector of Bangladesh, so they can better respond to the challenges of CC and their effects on health.

## Methods

### Design and setting

This community-based cross-sectional study was carried out in 2012 in seven purposely-selected districts of Bangladesh, namely Bagerhat, Borguna, Coxbazar, Faridpur, Khulna, Sathkhira and Shirajganj. These are known to be cyclone, flood and salinity prone and hence constitute the most vulnerable areas to climate change [25]. The total population of the seven districts is 19,228,598 individuals (census 2011). This survey was jointly conducted by the Climate Change and Health Promotion Unit (CCHPU) of the Ministry of Health and Family Welfare Bangladesh, Health Communication Network, Bangladesh, and the University of Newcastle, Australia.

### Sample size calculation

We calculated the sample size for this study based on the precision of the prevalence of peoples’ knowledge about CC. We assumed that approximately 50 % of people have some knowledge about climate change. To estimate this proportion with a 95 % confidence interval, 371 participants are required. We used a four-stage multilevel cluster sampling technique. Assuming a design effect of 2 for each stage, the total design effect is 16, and the required sample size is 371 × 16 = 5936. To allow for a 10 % non-response and for 1 % missing data, the required sample size is 6670. To evenly distribute the samples across districts we sampled 960 households from each of the seven districts, a total of 6720 households. In this study the knowledge of climate change was assessed only for the head of each household.

### Study population

The targeted study population was the total community of the seven vulnerable districts. Multistage sampling was used to select respondents for the study. We randomly selected four upazilas (sub-district) from each district as the primary sampling units. Then four unions (lowest administrative unit) per upazila (a total of 112 unions) were randomly selected, followed by 2 villages from each union. Thus a total of 224 villages were finally selected. The Bangladesh Bureau of Statistics (BBS) has standard Enumeration Areas (EA) and household listing for its census, which we used for EA and household selection. From each selected village, we randomly selected one EA and 30 households within each EA, using the BBS household numbers (total number of households =6720). From each household, we selected the head of the household as eligible participant, and obtained written consent or thumb impression. The consented participant provided information on other household members.

### Development of climate change knowledge-perception questionnaire

The main instrument used for this study was a pretested, structured interviewer-administered interview schedule. We reviewed literature on CC for developing questionnaires on CC and health knowledge, perception, attitude and behaviour measures in relation to CC. We also contacted experts in the field to check for availability of a valid tool to assess CC knowledge. All perception questions on CC or health or CC extreme events had responses using various Likert-type answers such as ‘Yes’, ‘No’ or ‘Did Not notice’, or categorical options. The final interview schedule comprised a set of 37 questions on various aspects of climate change and health issues. As a part of the questionnaire, we also developed an observation checklist of household and community characteristics used by the interviewers (Additional files 1 and 2).

### Measurement of outcome variables

The number of deaths from drowning and snake bites during extreme weather events was collected for the last 10 years preceding the interviews. The number of snake bites received by any household member during the previous 12 months was recorded. Prevalence of diarrhoea and pneumonia of children aged under five, and of Dengue fever and malaria in any household member, was collected and is presented elsewhere (unpublished observations by Kabir MI, Rahman MB, Smith W, Lusha MAF and Milton AH). Participants who indicated that they had heard of CC were asked for their explanation of CC, followed by questions about its causes. The knowledge score was obtained from this information. We also asked CC related specific questions to all the respondents to assess their perception of CC irrespective of their knowledge about CC.

### Data Collection

A structured draft questionnaire was developed and finalised after pre-testing in a similar rural setting. The final questionnaire was used to collect information on socio-demographic characteristics of the participants, and their knowledge and perception of climate change, extreme weather events and health. Other relevant information on health services and health education programmes at school was collected through face-to-face interviews.

A week-long training was provided to the data collectors. The trained 28 interviewers administered the questionnaire at household level under the direct supervision of 14 field supervisors over a two-week period. The quality-control team formed by the investigators monitored the performance of field personnel and supervisors through regular observation at the household level and regular checks of data for completeness. In 5 % of study participants, the quality control team independently repeated data collection. The investigators checked each filled-up questionnaire to ensure that no information was missing; any error detected was corrected immediately at the field, sometimes by revisiting the household, before it was entered into the computer.

### Statistical analysis

Collected data were entered into the computer and validated using the Statistical Package for Social Sciences (SPSS), version 21 by a series of logical and range checks, producing summary statistics and tables. Data were immediately copied onto the hard disks of two computers as soon as data verification was complete, and a copy was sent to the Principal Investigator at the University of Newcastle, Australia.

The summary statistics were reported as means with standard deviations (SD) for continuous variables, or as percentages with 95 % confidence intervals (CI) for categorical variables. Sociodemographic variables and participants’ responses were summarized and presented using frequency tables. Evidence of association between sociodemographic variables and knowledge of climate change was explored by cross-tabulation and measured using chi-square tests. Logistic regression models were applied to further explore the predictors of knowledge that were significant using bi-variate analysis. Where necessary, we also estimated dose–response relations using the ‘contrast’ post-estimation command after fitting a logistic regression in Stata (Stata 13 STATA Corporation, Texas, USA). We estimated the incidence densities of different events (e.g. death from drowning) by fitting intercept-only zero-inflated Poisson models using total number of household member as the offset. To adjust for the design effect of multistage sampling, we created sampling weights and incorporated them in the analyses using the ‘svy:’ command in Stata.

### Ethical approval and consent

The study protocol was approved by the Bangladesh Medical Research Council and by the Human Research Ethics Committee of the University of Newcastle, Australia (H2012-0163). Written informed consent (thumb impression in case of writing inability) was obtained from the heads of the household after the written information note was read out at the beginning of the interview.

## Results

A total of 6720 participants from 224 cluster villages in Bangladesh were interviewed. Each cluster contains 30 households in each of 56 unions within 28 upazilas in 7 coastal districts.. All participants were head of their households, comprising 6245 males (92.9 %) and 475 females (7.1 %). The mean age of the participants was 44.7 years with a standard deviation (SD) of 13.5 years. The mean duration of stay in the locality was 30 years (median 25, SD 15). The majority was in their middle age (45–60 years, 55.7 %), currently married (91.6 %), and had experienced extreme weather events (95.7 %) during their stay in the locality. As the study was conducted in coastal and rural Bangladesh the participants were mainly living in non-brick houses (91.3 %), and their main source of drinking water were either shallow (43.7 %) or deep (41.9 %) wells. Participants came largely from low socioeconomic backgrounds: only 9.6 % had post-secondary education or higher; the majority were day labourers or farmers (60 %), and only 10 % earned a monthly income above BDT 12000 (1 USD = 77 Bangladeshi Taka.) (Table 1).

**Table 1:** Characteristics o00f the participants (*n* = 6720)

Variable	Frequency (%)
Gender (Male)	6245 (92.9)
Age (Mean ± SD) in years	44.7 ± 13.5
Duration of stay in this locality (Mean, median, SD) in years	30, 25, ± 15
Education of Respondent	
No formal education	2923 (43.5)
Primary	2013 (30.0)
Secondary	1143 (17.0)
Higher Secondary	496 (7.4)
Graduate and above	145 (2.2)
Occupation of Respondent	
Farmer	1988 (29.6)
Day labourer	2047 (30.5)
Service holder	524 (7.8)
Small and medium business	1066 (15.9)
House wife	299 (4.5)
Fisherman	252 (3.8)
Unemployed	51 (0.8)
others	493 (7.3)
Type of House	
Kachca (Bamboo, Tree Leaves))	925 (13.7)
Pakka (Brick)	121 (1.8)
Tin (Corrugated sheet)	5211 (77.6)
others	462 (6.9)
Total household monthly income	
Income (In Bangladeshi Taka, 1 US$ = 80 BDT approx..)	
< 4000 BDT	1436 (21.4)
4000–8000 BDT	3485 (51.9)
8000–12000 BDT	1127 (16.7)
> 12000 BDT	672 (10)
Drinking water source	
Shallow Tube well	2936 (43.7)
Deep tube well	2822 (42.0)
Supply water	36 (0.5)
Untreated water	322 (4.8)
Treated water	524 (7.8)
Rain water	50 (0.7)
Others	30 (0.5)

Table 2 shows the responses of the participants on their climate change knowledge. The majority (*n* = 6720) of the research participants (54.2 %) acquired knowledge about CC from some source and 45.8 % had not heard of CC at all. Among the knowledgeable participants (*n* = 3645), the majority felt that CC was manifested in an increase in (83.2 %). More than half (53.9 %) attributed a change in the pattern/periodicity of rainfall to CC, 43.2 % felt a colder winter than usual cold, and more than one third saw CC in a higher incidence of cyclones or tidal waves (36.5 %). Some thought that frequent flooding (13.8 %) and water logging (8.7 %) are an effect of CC. When participants were asked about causes for CC (*n* = 3645), responses varied from deforestation (81 %) to rapid urbanization and changes in life style (1.9 %). More than half of the participants felt that CC was due to population growth (56.9 %). As many as 28.1 % believed that industrial effluents are one of the cause, 25.1 % chose black exhaust from vehicles, and 4.9 % attributed CC to excessive carbon emissions by the developed countries. In terms of the source of knowledge, television was the most commonly mentioned (55.6 %), followed by neighbours (54.8 %) and radio (39 %) (Table 2).

**Table 2:** Participants’ knowledge about climate change

Variable	Frequency (%)
Have you heard of what ‘Climate Change’ means from any source?	
Yes	3645 (54.2)
No	3073 (45.8)
What is the main source of your information on climate change?^a^	
Newspaper	416 (11.4)
Weekly magazine	51 (1.4)
Radio	1423 (39.0)
Television	2026 (55.6)
Neighbours	1999 (54.8)
Health workers	513 (14.0)
Teachers	164 (4.5)
Family members/Relatives	12 (0.33)
Imams of the mosque	1 (0.03)
NGO workers	5 (0.14)
Personal involvement in Training	1 (0.03)
Type of change in climate^a^	
Excessive Temperature	3033 (83.2)
Excessive cold	1575 (43.2)
Change of pattern of rainfall	1967 (53.9)
Frequent cyclone or tidal wave	1329 (36.5)
Frequent Flood	502 (13.8)
Water logging	317 (8.7)
Don’t know/Don’t understand	
Causes or reasons for climate change^a^	
Deforestation	2953 (81.0)
Industrial effluents	1023 (28.1)
Population Growth	2075 (56.9)
Black smoke of vehicles	914 (25.1)
Excessive carbon emission by the developed country	177 (4.9)
Rapid urbanization and changes in life style	71 (1.9)
Others	32 (0.9)

^a^(*n* = 3645) percentage total may add up to more than 100 % as multiple responses were permissible

We also asked all the participants CC-related questions, irrespective of their knowledge about CC, to explore their perception and awareness towards the influence of CC on human health and the environment (Table 3). Participants observed increased episodes of extreme weather events such as drought (85.5 %), cyclones/floods (61.3 %), and tidal waves (42.1 %) in the past 10 years preceding the study. Most of them (91.9 %) observed a change in rainfall pattern over the last 10 years. Although 54.2 % of participants had heard of CC, 94.5 % had observed changes in climate. Some of the participants perceived that the sea water level had increased (17.4 %), while 46.7 % had not noticed this.in the coastal areas. About 29 % of the total households were not living in the coastal area but they were also been asked the question.

**Table 3:** Participants’ perception and awareness towards influence of climate change on human health and the environment (*N* = 6716)

Influence of Climate Change	Yes N (%)	Unchanged^a^/ Not applicable^b^ N (%)	Did not notice N (%)
Increased episode of cyclone/flood in the last 10 years	4120 (61.3)	188 (2.8)^a^	0
Increased episode of tidal wave in the last 10 years	2823 (42.1)	467 (6.9)^a^	2846 (42.5)
Increased episode of drought in the last 10 years	5748 (85.5)	229 (3.4)^a^	139
Change in rainfall pattern in the last 10 years	6172 (91.9)	0	0
Change in sea water level in the last 10 years	1169 (17.4)	1920 (28.6)^b^	3140 (46.7)
Increased salinity of water in the last 10 years	3582 (53.3)	0	0
Scarcity of fresh water due to increase in salinity	5378 (80)	0	0
Increased health risk due to increase in salinity	4073 (60.6)	0	0
Reduced food crop production in the last 10 years	4761 (70.8)	0	0
Death from drowning in the last 10 years	132 (32, 95 % CI: 27 to 38)^c^	0	0
Death from snake bite in the last 10 years	26 (6.8, 95%CI: 3.5 to 13.2)^c^	0	0
Number of snake bite in the last year	207 (36, 95%CI:29 to 45)^c^		
Increased health care expenditure after extreme weather events	6572 (97.8)	0	0

^a^Unchanged is meant for the increased episodes of extreme weather events

^b^Not applicable means the households were not in coastal area

^c^Incidence densities per 100,000 per year

In addition to the environmental elements, participants’ perception of CC-related health effects was also limited. Most of the participants (80 %) believed there will be a scarcity of fresh water due to increase in salinity, as a majority (53.3 %) had observed increased water salinity in the past 10 years preceding the interview. About 61 % were aware of increasing health risk such as high blood pressure, pregnancy outcomes because of increase in salinity. In terms of food security, almost 71 % indicated that food crop production would be negatively affected. The incidence of deaths from drowning and snake bite during the extreme weather events of the last 10 years were 32 (95 % CI: 27 to 38) and 6.8 (95 % CI: 3.5 to 13.2), respectively, per 100,000 population per year. The incidence of snake bite for the year preceding the study period was 36 (95 % CI: 29 to 45). Almost all participants (97.8 %) believed health care expenditure increased after extreme weather events (Table 3).

Cross-tabulation was done to examine the association between sociodemographic variables and the knowledge of the participants on CC. Age, sex, education, occupation, monthly family income, nearby government health facility, and presence of any school in the survey area were significantly associated with the knowledge of participants (Table 4).

**Table 4:** Association between socio-demographic variables and knowledge of climate change

Variable	Knowledge of climate change			Statistics
	No (%)	Yes (%)	Total (%)	
Age-group				
Young (<30 years)	384 (51.7)	359 (48.3)	743 (100.0)	*χ* ^*2*^ *= 13.84*
Early adulthood (30 – 45 years)	1243 (46.0)	1458 (54.0)	2710 (100.0)	*df = 3*
Middle age (45 – 60 years)	929 (44.3)	1167 (55.7)	2096 (100.0)	*p = 0.003*
Old age (>60 years)	517 (43.9)	659 (56.1)	1176 (100.0)	
Total	3073 (45.8)	3643 (54.2)	6716 (100.0)	
Sex				
Male	2811 (45.0)	3430 (55.0)	6241 (100.0)	*χ* ^*2*^ *= 18.20*
Female	262 (55.1)	213 (44.9)	475 (100.0)	*df = 1*
Total	3073 (45.8)	3643 (54.2)	6716 (100.0)	*p < 0.001*
Education of Respondent				
No formal education	1571 (53.8)	1350 (46.2)	2921 (100.0)	*χ* ^*2*^ *= 334.70*
Primary	993 (49.4)	1018 (50.6)	2011 (100.0)	*df = 4*
Secondary	383 (33.5)	760 (66.5)	1143 (100.0)	*p < 0.001*
Higher Secondary	107 (21.6)	389 (78.4)	496 (100.0)	
Graduate and above	19 (13.1)	126 (86.9)	145 (100.0)	
Total	3073 (45.8)	3643 (54.2)	6716 (100.0)	
Occupation of Respondent				
Farmer	1025 (51.6)	961 (48.4)	1986 (100.0)	*χ* ^*2*^ *= 180.04*
Day labourer	1011 (49.4)	1034 (50.6)	2045 (100.0)	*df = 7*
Service holder	136 (25.9)	388 (74.1)	524 (100.0)	*p < 0.001*
Small and medium business	398 (37.3)	668 (62.7)	1066 (100.0)	
House wife	169 (56.5)	130 (43.5)	299 (100.0)	
Fisherman	126 (50.0)	126 (50.0)	252 (100.0)	
Unemployed	19 (37.2)	32 (62.8)	51 (100.0)	
others	189 (38.3)	304 (61.7)	493 (100.0)	
Total	3073 (45.8)	3643 (54.2)	6716 (100.0)	
Total household monthly income				
Income (cat)				
< 4000BDT	742 (51.8)	692 (48.2)	1434 (100.0)	*χ* ^*2*^ *= 71.96*
4000–8000 BDT	1653 (47.5)	1830 (52.5)	3483 (100.0)	*df = 2*
> 8000 BDT	678 (37.7)	1121 (62.3)	1799 (100.0)	*p < 0.001*
Total	3073 (45.8)	3643 (54.2)	6716 (100.0)	
Nearby government health facility				
District Hospital	127 (38.2)	205 (61.8)	332 (100.0)	*χ* ^*2*^ *= 68.79*
Upazila health complex	790 (39.0)	1235 (61.0)	2025 (100.0)	*df = 3*
Union health centre	1214 (49.6)	1233 (50.4)	2447 (100.0)	*p < 0.001*
Community clinic	942 (49.3)	970 (50.7)	1912 (100.0)	
Total	3073 (45.8)	3643 (54.2)	6716 (100.0)	
Any school in the survey area				
No	101 (66.9)	50 (33.1)	151 (100.0)	*χ* ^*2*^ *= 27.79*
Yes	2972 (45.3)	3593 (54.7)	6565 (100.0)	*df = 1*
Total	3073 (45.8)	3643 (54.2)	6716 (100.0)	*p < 0.001*

Education was significantly associated with CC knowledge in the univariate analyses. In the multivariable analysis, education, sex and availability of school in the area were significantly associated with knowledge of CC and its health impacts. A clear dose–response association was observed with educational level (P-for linear trend from logistic regression <0.001). Respondents from a locality with a school were three times more likely to have knowledge about CC and its impact on health than those who did not have a school in their locality (Table 5).

**Table 5:** Predictor of knowledge of climate change from logistic regression analyses

Predictor	Unadjusted odds ratio (95 % CI)	Adjusted odd Ratio (95 % CI)
Sex (Female)	0.61 (0.46–0.82)	0.71 (0.53–0.95)
Age	1.002 (0.995–1.008)	1.003 (0.99–1.001)
Education^a,b^		
No formal education	1	1
Primary	1.02 (0.73–1.43)	1.02 (0.72–1.46)
Secondary	2.06 (1.43–2.97)	2.03 (1.39–2.98)
Higher Secondary	3.15 (1.83–5.42)	3.06 (1.75–5.34)
Graduate and above	7.16 (3.17–16.19)	6.90 (3.07–15.48)
Availability of school in the locality	3.18 (1.32–7.66)	3.16 (1.32–7.54)

^a^ *P* < 0.001, for overall significance of the variable in the multivariable model

^b^ *P* < 0.001, for linear trends in the odds ratios

## Discussion

This study, to the best of our knowledge, is the first large-scale quantitative attempt to assess the knowledge and perceptions of the vulnerable communities in Bangladesh about CC and its impact on health. The findings provide important insights into what people think and believe from their experience at the grassroots level. The household participants were from the rural areas that are vulnerable to climate change [25]. Since we interviewed the heads of the households, most of the participants were male. The majority of participants were relatively poor and had little formal education with an agro-based livelihood, which is consistent with the national level data [26]. However, they have a clear perception about the change in climate and associated variability in temperature, rainfall, salinity and sea level, and the effect of these changes on their health. Knowledge and perception studies on CC and its associated health impact had been conducted in the Philippines [27], Vietnam [28], United States of America, Canada, and Malta [29], Nigeria [30], Nepal [31], Bangladesh [24], India [32], Australia [33], and China [34]. A previous cross-sectional study of two villages in Bangladesh had the limitation of generalizability of their findings regarding households’ perception of CC and human health risks [24]. In our study, we used a similar approach but our sample size was larger, more representative and better distributed geographically among vulnerable communities.

Out of 6720 participants, slightly more than fifty four percent had knowledge of ‘climate change’. Almost 46 % of the vulnerable community had not heard about CC, which might be a concern for the policy makers in health sector to provide adequate community level training on CC and its impact on health through the community clinics. The results are similar in two other studies conducted in LDCs, Nigeria [30] and Nepal [35], where 54 % and 51.3 %, respectively, of the participants reported they know about ‘climate change’. From our study, of those who had knowledge about CC, the majority named mass media (television, radio, newspaper etc.) and neighbours as their source. Very few people mentioned hearing about CC from teachers, Imams, health workers and non-governmental organization (NGO) workers. This indicates that mass media and discussions in the neighbourhood are important source of information. Opinion leaders such as teachers and imams of mosques, and service providers such as health workers and NGO workers need formal training on CC and its impact on health in the field level. There is a sectoral gap of engagement from the government system with health and environmental issues. This is consistent with the study conducted in Nepal [35]. Overall, our study highlights that mass media coverage, especially through community radio and television, is currently the major source of information about CC.

Most of the participants believed that there had been a change in climate in the 10 years preceding the interview. This perception is supported by data collected at national level on the climate in Bangladesh. Data from the Bangladesh Meteorological Department (BMD) for the periods of 1951–2011 show that for any ten-year period the temperature was higher and rainfall less predictable than in the preceding ten years [36]. Other countries such as Jamaica, Vietnam, and Nepal reported identical findings for their studies among a similar population [23, 28, 35]. With regard to causes or reasons for CC, most of our participants mentioned deforestation followed by population growth and industrial effluents. The other less scientific reasons given by the participants, such as black smoke of vehicles, could be due to their low level of education and less exposure to the scientific facts by virtue of their occupation. These results also supported by other studies carried out in Bangladesh and other LDCs such as Nigeria and small island countries such as Trinidad and Tobago [30, 37].

The perceptions of vulnerable people are also consistent with the national level findings. Annual maximum temperature over Bangladesh is increasing by 0.09 °C but annual minimum temperature is increasing by 0.10 °C. The rate of increase of the annual minimum temperature is higher than that of the annual maximum temperature [38]. The annual average rainfall over Bangladesh is increasing by about 10.6 mm per decade whereas monsoonal rainfall over Bangladesh is decreasing by 7.6 mm per decade [39]. The participants also had the perceptions that episodes of extreme weather events such as cyclones, floods, storm surges, heavy rains within a short time, and droughts had increased compared to ten years earlier. It has been observed from the BMD database that the frequency of cyclones has been increasing in every decade since the 1970s [40]. Bangladesh evidenced ten severe floods during the last 40 years. Every 4–5 years severe floods inundate about 60 % of the country. In 2007, two successive and damaging floods occurred in the same season [41]. It has been found that the frequency of heavy rainfall in June, July and August has increased over the last few decades [39].

With regard to the sea level rise, the perception of the coastal participants from Borguna, Bagerhat, Cox’s bazar, Khulna and Satkhira is supported by historic data on sea water level rise in three of the coastal districts: Bagerhat, Satkhira and Khulna. These data show that the mean annual water level has risen by 4.5 mm/year in about 20 % of the total area of these three districts, which is attributed to the global sea level rise associated with anthropological activities [42]. The perception of our participants regarding salinity intrusion and increase in water salinity is also supported by another evidence-based study [43].

Participants strongly agreed that agricultural food crop production had declined during the last 10 years, and population health had been adversely affected by CC in their villages. This has serious implications regarding food security and livelihood. The number of deaths from drowning and snake bites supports other community-based studies in Bangladesh [44, 45], although the incidence was higher in our study because of the increase in the number of extreme weather events. This finding has important implications for local level planning and policy development. Moreover, understanding the concerns of people about increased health care expenditure after the extreme weather events may help policy makers to develop and implement apprpriate adaptive measures for health adaptation.

In our study, heads of households with higher levels of education were more likely to be aware of CC than those with lower education levels. Other studies also reported that CC awareness most strongly depended on the respondent’s level of education [27, 31, 46]. In the course of educational development of Bangladesh the availability of schools in rural and remote areas was a major factor in students’ drop out and people’s education level. Our study suggests that schools play an important role for increasing knowledge on CC and associated adaptation issues.

Our study has several strengths. We surveyed a large number of households. Furthermore, the study recruited participants from an array of geographic locations known for their susceptibility to CC. This makes the findings from this study relevant to other CC-vulnerable areas of Bangladesh. A further strength is the collection of data with local context in association with how extreme weather events were experienced. The findings will be useful for developing strategies to induce individual and community behaviour change to better cope with CC and make adaptations to minimise health issues. The findings could be used as baseline for a future cohort study. There were a few limitations to the study, including the recall bias, as we relied on the previous experiences and subjective judgements of the head of the households. As 92.9 % of participants were male, female perception was not considered. Peoples’ perception on CC and its impact on health could not be checked against real-time data. Such data could be collected in future to allow estimating the accuracy of community perceptions.

## Conclusion

The knowledge level of the study participants on CC was more than our assumption (54 %) and the perception of vulnerable communities on changing climatic factors and their impacts on health was higher (94 %). The public health sector needs to be engaged more in primary health care by training health workers at field level to address CC and health issues. Level of education is an influential factor to understand CC and its impact on health. Based on these key findings, policy makers can develop effective communication strategies for the vulnerable communities in Bangladesh and similar LDCs to protect human health from the adverse effect of CC. To protect future generations child-centred educational intervention at school level on CC and health adaptation could be explored by further research [47].

## Additional files

Additional file 1: Survey Questionnaire in Local laguage Bengali. (PDF 2502 kb) Additional file 2: Survey Questionnaire English version. (PDF 51 kb)

## Abbreviations

AR5: The fifth Assessment Report of IPCC
BBS: Bangladesh Bureau of Statistics
BDT: Bangladeshi Taka
BMD: Bangladesh Meteorological Department
CC: Climate change
CCHPU: the Climate Change and Health Promotion Unit of the Ministry of Health and Family Welfare
IPCC: Intergovernmental Panel on Climate Change
LDC: Least Developed Countries
USD: United States Dollar
WHO: World Health Organization
