# Supplementary material for: Knowledge and perception about climate change and human health: findings from a baseline survey among vulnerable communities in Bangladesh
Source: BMC Public Health. 2016 Mar 15;16:266. doi: 10.1186/s12889-016-2930-3 (PMC4791871; doi:10.1186/s12889-016-2930-3)
Supplement: Additional file 1: — Survey Questionnaire in Local laguage Bengali. (PDF 2502 kb) [file 12889_2016_2930_MOESM1_ESM.pdf]

Sample ID:					

Risk Reduction and Adaptive Measures in the Context of Climate Change Impact on Health Sector in Bangladesh

Baseline Survey Questionnaire

July - 2012

Implemented by

Climate Change and Health Promotion Unit (CCHPU)
Under
Ministry of Health & Family Welfare
14/2, Topkhana Road, Ansari Bhaban, Dhaka-1000.
Bangladesh

In Collaboration with
The University of New Castle, Australia and
Health Communication Network

Baseline Survey Questionnaire

Risk Reduction and Adaptive Measures in the Context of Climate Change Impact on Health Sector in Bangladesh

তথ্যসংগ্রহের তারিখ --- / --- / ২০১১

১. উত্তরদাতার নাম : .... কোড নং | | | |

২. জেলার নাম : .... কোড নং | | | |

৩. উপজেলার নাম : .... কোড নং | | | |

৪. ইউনিয়ন/ ওয়ার্ড : .... কোড নং | | | |

৫. গ্রাম/ মহল্লা/ ব্লক : .... কোড নং | | | |

৬. তথ্য সংগ্রহকারীর নাম ও স্বাক্ষর : ....

১.পরিবারের সদস্যদের নামের তালিকা :

(পরিবার প্রধানের নাম দিয়ে শুরু করুন)

ক্রমিক নং	পরিবারের সদস্যদের নাম	পরিবার প্রধানের সাথে সম্পর্ক	লিঙ্গ	বয়স/মাস*	বৈবাহিক অবস্থা	শিক্ষাগত যোগ্যতা	পেশা
১	২	৩	৪	৫	৬	৭	৮
১.							
২.							
৩.							
৪.							
৫.							
৬.							
৭.							
৮.							
৯.							
১০.							
১১.							

(৩, ৪, ৬, ৭, ৮ নং কলামের জন্য নিম্নের কোড ব্যবহার করুন)

* (শুধুমাত্র (০- ৫৯) মাসের শিশুদের জন্য মাসে উল্লেখ করুন)

পরিবার প্রধানের সাথে সম্পর্ক :	লিঙ্গ:	বৈবাহিক অবস্থা :	শিক্ষাগত যোগ্যতা:	পেশা:
১. পরিবার প্রধান<br>২. স্বামী বা স্ত্রী<br>৩. পুত্র বা কন্যা<br>৪. পিতা-মাতা<br>৫. অন্যান্য	১. পুরুষ<br>২. মহিলা	১. অবিবাহিত<br>২. বিবাহিত<br>৩. তালাকপ্রাপ্তা<br>৪. বিধবা<br>৫. বিপত্নীক<br>৬. অন্যান্য	১. আনুষ্ঠানিক কোন শিক্ষা নাই<br>২. প্রাথমিক স্তর (১ম - ৫ম শ্রেনী পর্যন্ত)<br>৩. মাধ্যমিক স্তর (৬ষ্ঠ - ১০ম শ্রেনী পর্যন্ত)<br>৪. এসএসসি / এইচএসসি / দাখিল / আলিম<br>৫. স্নাতক বা এর উপর	১. কৃষিজীবী<br>২. দিনমজুর<br>৩. চাকুরিজীবি<br>৪. ক্ষুদ্র ব্যবসায়ী<br>৫. গৃহিণী<br>৬. বেকার<br>৭. ছাত্র<br>৮. জেলে<br>৯. অন্যান্য

পারিবারিক অবস্থা :

২. আপনার বাড়িতে মোট ঘরের সংখ্যা কত ?

....

৩. আপনার পরিবারের সদস্যদের মোট মাসিক গড় আয় কত টাকা?
(একাধিক উপার্জনকারী থাকলে সবার আয়সহ নিতে হবে)

....

দুর্যোগ সম্পর্কিত প্রশ্ন :

৪. এই এলাকায় আপনি কত বছর যাবৎ বসবাস করছেন ?

....

৫. এখানে অবস্থানকালে আপনি কি কোনো ধরনের প্রাকৃতিক দুর্যোগের মোকাবেলা করেছেন ?
১. হ্যাঁ
২. না (উত্তর যদি হ্যাঁ হয় তাহলে পরবর্তী প্রশ্ন করবেন / উত্তর যদি না হয় ১০ নং প্রশ্নে যাবেন)

৬. আপনি কী ধরনের প্রাকৃতিক দুর্যোগের মোকাবেলা করেছেন ? (উত্তর একাধিক হতে পারে)
১. বন্যা
২. খরা
৩. ঘূর্ণিঝড়
৪. জলোচ্ছ্বাস
৫. ভুমিকম্প
৬. নদী ভাঙ্গন
৭. অন্যান্য

৭. দশ বছরের মধ্যে প্রাকৃতিক দুর্যোগের কারণে আপনি কি ঘরছাড়া হয়েছেন ?
১. হ্যাঁ
২. না (উত্তর যদি হ্যাঁ হয় তাহলে পরবর্তী প্রশ্ন করবেন / উত্তর যদি না হয় ১০ নং প্রশ্নে যাবেন)

৮. এ ক্ষেত্রে কতবার ঘরছাড়া হয়েছিলেন ?

....

৯. শেষবার কত দিনের জন্য ঘরছাড়া হয়েছিলেন ?

....

১০. আপনি কি আবহাওয়া / জলবায়ু পরিবর্তনের কথা শুনেছেন ?
১. হ্যাঁ
২. না (উত্তর যদি হ্যাঁ হয় তাহলে পরবর্তী প্রশ্নে করবেন / উত্তর যদি না হয় ১৪ নং প্রশ্নে যাবেন)

১১. আপনি এটা কোথা থেকে শুনেছেন? (উত্তর একাধিক হতে পারে)
১. সংবাদপত্র
২. ম্যাগাজিন
৩. রেডিও
৪. টেলিভিশন
৫. প্রতিবেশী
৬. স্বাস্থ্যকর্মী
৭. স্কুল শিক্ষক
৮. অন্যান্য (উল্লেখ করুন)..............

১২. জলবায়ুর ক্ষেত্রে কী ধরনের পরিবর্তন হয়েছে আপনি মনে করেন ? (উত্তর একাধিক হতে পারে)

১. অতিমাত্রায় গরম
২. অতিমাত্রায় শীত
৩. বৃষ্টিপাতের ধরনের পরিবর্তন
৪. সাইক্লোন বা ঘুর্নিঝড়
৫. ঘন ঘন বন্যা
৬. জলাবদ্ধতা
৭. জানি না / বুঝি না

১৩. কী কী কারনে জলবায়ুর পরিবর্তন হচ্ছে বলে আপনি মনে করেন ? (উত্তর একাধিক হতে পারে)

১. গাছপালা নিধন
২. কলকারখানার বিষাক্ত ধোঁয়া ও বর্জ্য
৩. জনসংখ্যা বৃদ্ধি
৪. যানবাহনের কালো ধোঁয়া
৫. বায়ুমন্ডলে উন্নত বিশ্বের অধিক মাত্রায় কার্বন নিঃসরন
৬. দ্রুত অধিক নগরায়ন ও জীবনযাত্রার পরিবর্তন
৭. অন্যান্য (উল্লেখ করুন)...............

১৪. গত দশ বছরে আপনার এলাকায় বন্যার / ঘুর্নিঝড়ের পরিমাণ : (ধারনা/অনুমান)

১. কমেছে
২. বেড়েছে
৩. অপরিবর্তিত

১৫. গত দশ বছরে আপনার এলাকায় জলোচ্ছ্বাসের ঘটনা (ধারণা/ অনুমান) (প্রযোজ্য হলে)

১. কমেছে
২. বেড়েছে
৩. অপরিবর্তিত
৪. লক্ষ করিনি

১৬. গত দশ বছরে আপনার এলাকায় খরার পরিমাণ (ধারণা/ অনুমান)

১. কমেছে
২. বেড়েছে
৩. অপরিবর্তিত
৪. লক্ষ করিনি

১৭. গত দশ বছরে আপনার এলাকায় বৃষ্টিপাতের পরিমাণের কোন পরিবর্তন লক্ষ করেছেন ?

১. হ্যাঁ
২. না

১৮. গত দশ বছরে আপনার পরিবারের কেউ পানিতে ডুবে মারা গিয়েছে কিনা ?

১. হ্যাঁ
২. না (উত্তর যদি হ্যাঁ হয় তাহলে পরবর্তী প্রশ্নে করবেন/উত্তর যদি না হয় প্রশ্ন নং ২০এ যাবেন)

১৯. কত জন পানিতে ডুবে মারা গিয়েছে ?

....

২০. গত দশ বছরে আপনার পরিবারের কেউ সাপের কামড়ে মারা গিয়াছে কি না ?

১. হ্যাঁ
২. না

২১. গত এক বছরে আপনার পরিবারের কেউ সাপের কামড়ে আক্রান্ত হয়েছে কি না ?

১. হ্যাঁ
২. না

২২. গত দশ বছরে আপনার এলাকায় সমুদ্রের পানির উচ্চতার কোন পরিবর্তন লক্ষ করেছেন ?
(প্রযোজ্য হলে প্রশ্ন করুন ,না বা জানি না হলে প্রশ্ন নং ২৫ এ যাবেন)
১. হ্যাঁ
২. না
৩. জানি না

২৩. আপনি কি মনে করেন সমুদ্রের পানির উচ্চতা বৃদ্ধি পেলে জীবন ও জীবিকার ঝুঁকি বেড়ে যেতে পারে ?
১. হ্যাঁ
২. না

২৪. আপনি কি মনে করেন এ কারণে আপনি ও আপনার পরিবার ক্ষতিগ্রস্থ হবেন ?
১. হ্যাঁ
২. না

২৫. গত দশ বছরে আপনাদের এলাকার পানির লবণাক্ততা বেড়েছে কিনা ?
১. হ্যাঁ
২. না

২৬. আপনি কি মনে করেন লবণাক্ততা বৃদ্ধির কারণে মিঠা পানির সমস্যা হতে পারে ?
১. হ্যাঁ
২. না

২৭. আপনি কি মনে করেন যে লবণাক্ততা বৃদ্ধির কারনে স্বাস্থ্যের কোন পরিবর্তন হচ্ছে ?
১. হ্যাঁ
২. না

২৮. গত দশ বছরে আপনাদের এলাকার ফসলের উৎপাদন কমেছে কিনা ?
১. হ্যাঁ
২. না (উত্তর যদি হ্যাঁ হয় তাহলে পরবর্তী প্রশ্ন করবেন / উত্তর যদি না হয় প্রশ্ন নং ৩০ এ যাবেন)

২৯ ফসলের উৎপাদন হ্রাসের কারণ কি বলে আপনি মনে করেন ? (উত্তর একাধিক হতে পারে)
১. বন্যা
২. খরা/ অনাবৃষ্টি
৩. অতিবৃষ্টি
৪. লবণাক্ততা বৃদ্ধি
৫. রাসায়নিক সারের অপব্যবহার
৬. কীটনাশকের অপব্যবহার
৭. অন্যান্য (উল্লেখ করুন)...............

স্বাস্থ্যসেবা সম্পর্কিত তথ্যাবলি:

৩০. সাধারনত স্বাস্থ্যসেবা পাওয়ার জন্য আপনি কোথায় যান?
১. সরকারি হাসপাতাল
২. কমিউনিটি ক্লিনিক
৩. প্রাইভেট ক্লিনিক
৪. এনজিও স্বাস্থ্যসেবা কেন্দ্র
৫. গ্রাম্য ডাক্তার
৬. ফার্মেসি
৭. অন্যান্য (উল্লেখ করুন)...............

৩১. নিকটস্থ স্বাস্থ্যকেন্দ্রের সেবাপ্রদানকারীকে প্রয়োজনের সময় পাওয়া যায় কি না ?
১. হ্যাঁ
২. না

৩২. গত বারো মাসে আপনার পরিবারের কোন সদস্য নিম্নোক্ত রোগে আক্রান্ত হয়েছে কিনা ?

রোগের নাম	উত্তর	আক্রান্তের সংখ্যা
ডেঙ্গু	হ্যাঁ / না	
ম্যালেরিয়া	হ্যাঁ / না	

৩৩. ডেঙ্গু/ ম্যালেরিয়া কিভাবে ছড়ায়?

১. মশার কামড়ে
২. দূষিত পানি
৩. দূষিত খাবার
৪. মল-মূত্রের মাধ্যমে
৫. অন্যান্য (উল্লেখ করুন)..............

৩৪. গত বারো মাসে মধ্যে আপনার পরিবারের কোন শিশু ডায়রিয়া বা নিউমোনিয়ায় আক্রান্ত হয়েছে কিনা ?
(শুধুমাত্র (০- ৫৯) মাসের শিশুদের জন্য প্রযোজ্য)

রোগের নাম	উত্তর	শিশুর সংখ্যা
ডায়রিয়া	হ্যাঁ / না	
নিউমোনিয়া	হ্যাঁ / না	

৩৫. গত এক মাসের মধ্যে আপনার পরিবারের কোন শিশু ডায়রিয়া বা নিউমোনিয়ায় আক্রান্ত হয়েছে কিনা ?
(শুধুমাত্র (০- ৫৯) মাসের শিশুদের জন্য প্রযোজ্য)

রোগের নাম	উত্তর	শিশুর সংখ্যা
ডায়রিয়া	হ্যাঁ / না	
নিউমোনিয়া	হ্যাঁ / না	

৩৬. পরিবারের সদস্যদের স্বাস্থ্যসুরক্ষার জন্য গত ১২ মাসে আপনি কত টাকা ব্যয় করেছেন?
(হাসপাতালে ভর্তি,ঔষধ কেনা,ডাক্তার দেখানো , ল্যাব পরীক্ষা করা ইত্যাদি বাবদ)

টাকার পরিমাণ..

৩৭. আপনি কি মনে করেন যে, স্বাস্থ্যসুরক্ষার খরচ দুর্যোগের পর বেড়ে যায়?

১. হ্যাঁ
২. না

গবেষণাকর্মে সহযোগিতার জন্য আপনাকে ধন্যবাদ

সুপারভাইজারের নাম ও স্বাক্ষর ....

বেইজ লাইন সার্ভের কমন চেকলিস্ট

উত্তরদাতার নাম- ________ কোড- ____

জেলা কোড- ____ উপজেলা কোড - ____ ইউনিয়ন কোড - ____ গ্রামের কোড- ____

(জরিপকারী পর্যবেক্ষন করুন এবং টিক চিহ্ন দিন)

১. বাড়ির ছাদের ধরন কী? (মূল ঘরের)
- ক. কাঁচা
- খ. পাকা
- গ. টিন
- ঘ.অন্যান্য

২. বাড়িতে বিদ্যুৎ সংযোগ আছে কি? (সৌর বিদ্যুৎ সহ)
- ক. হ্যাঁ
- খ. না

৩. খাবার পানির প্রধান উৎস কী?
- ক. অগভীর নলকূপ
- খ. গভীর নলকূপ
- গ.পাইপের মাধ্যমে সরবরাহকৃত পানি
- ঘ. অপরিশোধিত পুকুরের পানি
- ঙ. পরিশোধিত পুকুরের পানি
- চ. বৃষ্টির পানি
- ছ. অন্যান্য

৪. নিকটস্থ সরকারি হাসপাতাল কোনটি?
- ক. জেলা সদর হাসপাতাল
- খ. উপজেলা স্বাস্থ্য কমপ্লেক্স
- গ. ইউনিয়ন স্বাস্থ্যকেন্দ্র
- ঘ. কমিউনিটি ক্লিনিক
- ঙ. অন্যান্য (নির্দিষ্ট করে লিখুন)-----------

৫. জরিপ এলাকার নিকটে কোন স্কুল আছে কি? [থাকলে স্কুলের নাম ও ধরণ (প্রাথমিক/মাধ্যমিক/উচ্চ মাধ্যমিক) উল্লেখ করুন]
- ক. হ্যাঁ
- খ. না ....

৬. সেই স্কুলে স্বাস্থ্য শিক্ষার ওপর কোন কার্যক্রম আছে কি?
- ক. হ্যাঁ
- খ. না

৭. জরিপ এলাকায় কোন কমিউনিটি ক্লিনিক আছে কি? (থাকলে কমিউনিটি ক্লিনিকের নাম লিখুন)
- ক. হ্যাঁ
- খ. না ....

৮. বিদ্যালয় ও কমিউনিটি ক্লিনিকের মধ্যে কোন যোগাযোগ বা সম্পর্ক আছে কি?
- ক. হ্যাঁ
- খ. না

৯. স্থানীয়ভাবে সরকারী/ বেসরকারী উদ্যোগে মশক নিধন কার্যক্রম পরিচালিত হয় কি না?
- ক. হ্যাঁ
- খ. না

১০. উত্তরদাতার -
- ক. ওজন মাপুন ____ কেজি খ. উচ্চতা মাপুন ____ সে.মি

১১. উত্তরদাতার পরিবারে ২(দুই) বছরের নিচে কোন শিশু আছে কি না-
- ক. হ্যাঁ
- খ. না

১২. উত্তর হ্যাঁ হলে শিশুর (একাদিক শিশু থাকলে সবচেয়ে বেশী বয়সী শিশুর ওজন নিন)
- ক. ওজন মাপুন ____ কেজি খ. উচ্চতা মাপুন ____ সে.মি

তথ্য সংগ্রহকারীর নাম ও স্বাক্ষর : ---------------------- সুপারভাইজারের নাম ও স্বাক্ষর :-------------------
