# Supplementary material for: Knowledge and perception about climate change and human health: findings from a baseline survey among vulnerable communities in Bangladesh
Source: BMC Public Health. 2016 Mar 15;16:266. doi: 10.1186/s12889-016-2930-3 (PMC4791871; doi:10.1186/s12889-016-2930-3)
Supplement: Additional file 2: — Survey Questionnaire English version. (PDF 51 kb) [file 12889_2016_2930_MOESM2_ESM.pdf]

Sample ID:					

Risk Reduction and Adaptive Measures in the Context of Climate Change Impact on Health Sector in Bangladesh.

Baseline Survey Questionnaire

2012

Implemented by
Climate Change and Health Promotion Unit (CCHPU)
Under
Ministry of Health & Family Welfare
14/2, Topkhan Road, Ansari Bhaban, Dhaka-1000.
Bangladesh

In Collaboration with
The University of New Castle, Australia and
Health Communication Network.

BASELINE SURVEY QUESTIONNAIRE

Risk Reduction and Adaptive Measures in the Context of Climate Change Impact on Health Sector in Bangladesh.

Survey Date:.....................

1. Participant Name : .... Code No. | | |

2. District Name : .... Code No. | | |

3. Upozila Name : .... Code No. | | |

4. Union/Word : .... Code No. | | |

5. Village/Moholla/Block : .... Code No. | | |

6. Surveyor Name and Signature :....................................

1. Name of the Family members:
   (Start from head of the Family)

Sl. No.	Name of the Family members	Relation with the Family Head	Gender	Age/ Month	Marital Status	Education Qualification	Occupation
1	2	3	4	5	6	7	8
1.							
2.							
3.							
4.							
5.							
6.							
7.							
8.							
9.							
10.							
11.							

(Use following code for column no. 3, 4, 6, 7, and 8)
* (Use Month Only for (0-59) monthly for Children aged less than 5 years of age)
[You will need to ensure that the surveyor specifies months or years for each entry]

Relation with the Family Head	Gender	Marital Status	Educational Qualification	Occupation
**Column 3**	**Column 4**	**Column 6**	**Column 7**	Column 8
1. Head of the family	1. Male	1. Unmarried	1. No Institutional education	Farmer
2. Husband/Wife	2. Female	2. Married	2. Primary level (class 1-5)	Day Labour
3. Son/Daughter		3. Divorcee	3. Secondary Level (Class6-10)	Service holder
4. Father-Mother		4. Widow	4. SSC/HSC/ Dakhil/ Alim	Small businessman
5. Others		5. Bipotnik	5. Graduation & above	Housewife
		6. Others	6. Other/ Not applicable	Unemployed
			7. Student	
			8. Fishermen	
			9. Others	

Family Condition:

2. Total number of houses at your home?

   ....

3. Total average monthly income?

   ....

Disaster Related Question :

4. How long have you been living this area?

   ....

5. Have you faced any sort of Natural calamity such as flood, drought or cyclone?
   I. Yes
   II. No
   (If yes then go to the next question/If no then jump to the Q # 10)

6. What type of calamity you have faced
   (Multiple answer acceptable)
   I. Flood
   II. Drought
   III. Cyclone
   IV. Tidal wave
   V. River erosion
   VII. Other

7. Have you become homeless due to natural calamity during the past 10 years?
   I. Yes
   II. No
   (If 'Yes' then go to the next question/If 'No' then jump to the Q # 10)

8. How many times have you have been homeless due to a natural calamity in the past 10 years?

   ....

9. How many days were you homeless the last time?

   ....

10. Have you heard about Weather/Climate change?
    I. Yes
    II. No
    (If yes then go to the next question/If no then jump to the Q # 14)

11. Where did you hear about Climate change?
    (Multiple answer acceptable)
    I. Newspaper
    II. Magazine
    III. Radio
    IV. Television
    V. Neighbour
    VI. Health worker
    VII. School Teacher

VIII. Others (Please mention)

12. What type of change to the climate do you think has happened?
    (Multiple answer acceptable)
    I. Excessive Temperature
    II. Excessive Cold
    III. Change of pattern of precipitation
    IV. Cyclone/Tidal wave
    V. Frequent flood
    VI. Water logging
    VII. Don't know/Don't understand

13. What do you think are the possible reasons for climate change?
    (Multiple answers acceptable)
    I. Deforestation
    II. Industrial effluent
    III. Population growth
    IV. Black smoke of vehicles
    V. Excessive carbon emission to the atmosphere by developed country.
    VI. Quick urbanization & change of livelihood
    VII. Others (Specify)

14. What is your opinion about the extent of Floods andCyclones during last 10 years?
    I. Have decreased
    II. Have increased
    III. Unchanged

15. what is your opinion about the extent of tidal waves in you locality during last 10 years?
    I. Have decreased
    II. Have increased
    III. Unchanged
    IV. Didn't notice

16. What is your opinionabout the extent of drought in last 10 years?
    I. Have decreased
    II. Have increased
    III. Unchanged
    IV. Didn't notice

17. Have you notice any change in precipitation during the last 10 years?
    I. Yes
    II. No

18. Have any family member drowned during last 10 years?
    I. Yes
    II. No
    (If 'yes' then go to the next question/If no then jump to the Q # 20)

19. How many drowned?

    ....

20. Have any family members been attacked by snake biting in last 10 years?
I. Yes

II. No

21. Have any family members died by Snake biting in last 10 years?
    I. Yes
    II. No

22. Have you noticed any changed in sea water level in last 10 years?
    I. Yes
    II. No
    II. Don't know

23. Do you think risk of life's livelihood can increase if sea water level increases?
    I. Yes
    II. No

24. Do you think you and your family would be affected by increasing sea water levels?
    I. Yes
    II. No

25. Has water salinity increased in your locality in the last 10 years?
    I. Yes
    II. No

26. Do you think increases in salinity might affect fresh water?
    I. Yes
    II. No

27. Do you believe human health is deteriorating due to increases in water salinity?
    I. Yes
    II. No

28. Has crop production in your area diminished during the last 10 years
    I. Yes, go to Q29
    II. No, go to Q 30

29. In your opinion what are the possible causes of the reduction in crop production?
    I. Flood
    II. Drought
    III. Excessive rain
    IV. Increase in Salinity
    V. Improper use of chemical fertilizer
    VI. Improper use of pesticides
    VII. Others (Please specify) ______________________________

30. What is your closest place of healthcare service?
    I. Govt. Hospital
    II. Community Clinic
    III. Private Clinic
    IV. NGO Healthcare centre
    V. Village Doctor
    VI. Pharmacy
    VII. Other (Specify Please) ______________________________________

31. Are service providers of nearby health centre available when necessary?
    I. Yes
    II. No

32. Have any family members been attacked by any of the following diseases during last 12 months?

Name of Disease	Ans.	Number of attacked
Dengue fever	Yes/No	
Malaria	Yes/No	

33. How does Dengue/Malaria spread?
    I. Mosquito biting
    II. Polluted water
    III. Contaminated Food
    IV. Through Stool - urine
    V. Other (Specify) ____

34. Have any of your children (aged 59 months or younger) been attacked by diarrhea/Pneumonia during the last 12 months?

Name of Disease	Ans.	Number of attacked
Diarrhoea	Yes/No	
Pneumonia	Yes/No	

35. Have any of your children (aged 59 months or younger) been attacked by diarrhea or pneumonia in the last 1 month?

Name of Disease	Ans.	Number of attacked
Diarrhoea	Yes/No	
Pneumonia	Yes/No	

36. How much money did you spend for keeping good health of family member(s)

    Total Expenditure...

37. Do you feel healthcare expenditure increases after disaster?
    I. Yes
    II. No

? Space for other comments

Thank you for your cooperation in research

Name of the Supervisor and Sign ....

Common Checklist on Baseline survey

Participant Name: ______ Code No. ______

District Code:		Upozilla Code:		Union Code:		Village Code:	

rveyor to complete this section by tick (√) the

1. Type of Roof ?
   I. Kaacha
   II. Pucca
   III. Tin
   IV. Other
2. Electricity ?
   I. Yes
   II. No
3. Source of Drinking Water?
   I. Shallow
   II. Deep Tubewell
   III. Supplied water through Pipe
   IV. Crude pond water
   V. Refined Pond water
   VI. Rain water
   VII. Other
4. Govt. Hospital nearby? (define nearby???)
   I. Dist. Hospital
   II. Upazilla Health Complex
   III. Union Health Center
   IV. Comm. Clinic
   V. Other (Specify)
5. School in the Survey Area?
   (Specify name and type)
   I. Yes
   II. No
6. Health Education Program in this school?
   I. Yes
   II. No
7. Community Clinic in the Survey Area?
   (If Yes then Name)
   I. Yes
   II. No
8. Exchange/Communication between School and Community Clinic?
   I. Yes
   II. No
9. Mosquito Crash Program locally?
   I. Yes
   II. No
10. Participant
    I. Weight ______ Kg II. Height ______ cm III Age ______ in months
11. Any children below 2 yrs
    I. Yes
    II. No
12. If 'yes' then take the weight (If more than 1 then take weight of Elder child)?
    I. Weight ______ Kg II. Height ______ cm III Age in months
